# Coffee consumption and risk of myocardial infarction: a dose-response meta-analysis of observational studies

**DOI:** 10.18632/oncotarget.23947

**Published:** 2018-01-04

**Authors:** Long Mo, Wei Xie, Xiaoqun Pu, Dongsheng Ouyang

**Affiliations:** ^1^ Department of Cardiology, Xiangya Hospital of Centre South University, Changsha, Hunan Province, PR China; ^2^ Institute of Genetic Pharmacology, Xiangya School of Medicine, Centre South University, Changsha, Hunan Province, PR China

**Keywords:** coffee consumption, myocardial infarction, risk factor, dose-response, meta-analysis

## Abstract

**Background:**

Previous epidemiological studies have provided inconsistent conclusions on the effect of coffee consumption in the development of myocardial infarction (MI). The aim of the study was to evaluate the influence of coffee consumption and its potential dose-response patterns on the risk of developing MI.

**Materials and Methods:**

Three databases were searched for evidence of eligible studies. A random-effects model was used to pool the fully adjusted odds ratios (ORs) and the corresponding 95% confidence intervals (CIs). Dose-response analysis was performed to show the effect of each cup increased in daily coffee drinking on the risk of MI.

**Results:**

Seventeen studies involving 233,617 participants were included in our study. The association between coffee consumption and risk of MI did not show statistical significance when pooling the outcome data for the coffee consumption categories of 1~2 vs. < 1 cup per day (OR = 1.06, 95% CI: 0.94–1.19) and 2~3 vs. < 1 cup per day (OR = 1.07, 95% CI: 0.94–1.23). Compared with < 1 cup, daily drinking of 3~4 cups and > 4 cups of coffee were significantly associated with the risk of MI, and the pooled ORs (95% CIs) were 1.40 (1.11–1.77) and 1.48 (1.22–1.79), respectively. The dose–response analysis showed a “J–shaped” curve relationship of the risk of MI with coffee consumption.

**Conclusions:**

Daily drinking of more than three cups of coffee was associated with a significantly increased risk of MI. This positive association was only found in men but not in women. The impact of gender on this association should be further evaluated.

## INTRODUCTION

Coffee is one of the world's most popular beverages, and it has been implicated in the development of cardiovascular diseases (CVDs) [1, 2]. As the coffee exposure is widespread and the incidence of CVDs is high in the western countries, thus, even a weak association between coffee consumption and incidence of CVDs would result in an enormous impact among the population. Because coffee has potential adverse effects on blood pressure [3], arrhythmia [4], blood cholesterol [5], coronary heart disease [6], and levels of homocysteine [7], we have reason to believe that coffee drinking might increase the risk of myocardial infarction (MI). Moreover, an acute effect of coffee drinking on the consequent activation of the sympathetic nervous system has also been suggested, which might trigger an MI [8]. However, the possible relationship between coffee consumption and the risk of MI have been inconsistent in the epidemiological studies [9–12]. A recent meta-analysis conducted by Brown et al. [13] indicated that coffee consumption reduces the risk of mortality after acute MI. Moreover, an animal study [14] showed that caffeine, a main component of coffee, could protect myocardium from myocardial ischemia/reperfusion injury in the rats by inhibiting inflammation and apoptosis.

Although previous meta-analyses have concluded the association between coffee drinking and incidence of coronary heart disease [6] and overall CVD [15], no meta-analysis has been conducted to evaluate this association specific for the risk of MI. As the current literature reported different results on the role of coffee consumption and risk of MI, we performed a dose-response meta-analysis to review current evidence and make a quantitative assessment on the association of dietary consumption of coffee and the risk of MI in the observational studies.

## RESULTS

### Study search and selection

**Figure 1:**
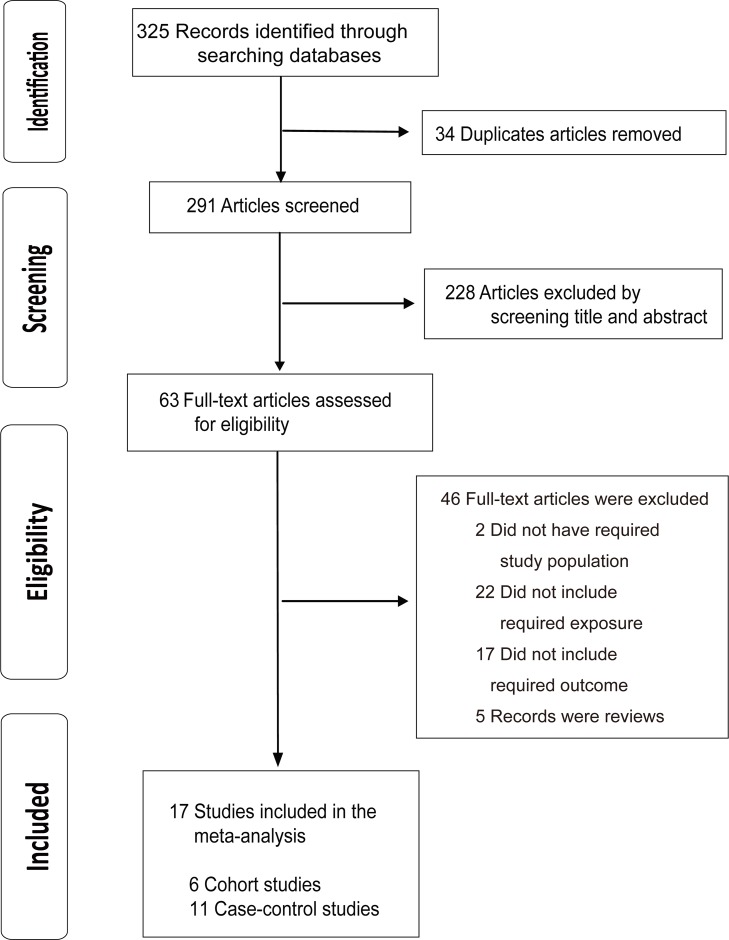
Flowchart for the selection of eligible studies

The selection process of eligible studies is shown in detail in a flow diagram (Figure 1). In the initial search, a total of 325 records were identified through searching the databases. After removing duplicates, 291 articles were screened for further judgment and evaluation. 228 articles were excluded by browsing the titles and the abstracts. Finally, our meta-analysis included seventeen studies, including 6 cohort studies [16–21] and 11 case-control studies [11, 22–31].

### Characteristics of included studies

The characteristics of each included article were shown in Supplementary Table 3. Twelve studies were performed in Europe, four in United States and one in Costa Rica. Six studies included both men and women, six studies included only men and five studies included only women. The sample size ranged from 310 to 101,774, involving 233,617 participants and 10,510 patients with MI. Only three studies included participants with mean baseline age of 60 years or above. Seven studies used a food-frequency questionnaire (FFQ) for assessment of coffee consumption, and other 10 studies used a self-administered questionnaire. Twelve studies used all types of coffee intake as the exposure factor, one study used caffeinated coffee intake, one study used decaffeinated coffee intake, and three studies used both caffeinated coffee intake and decaffeinated coffee intake. Potential confounding factors were controlled for in most of our included studies. The NOS scores of each included study are shown in Supplementary Table 3 and in detail in the supplementary materials (Supplementary Table 1 and Table 2). As a whole, the quality of the included studies was relatively good (7 points for six studies, 8 points for two studies, and 9 points for nine studies).

### Pooled analysis: coffee consumption and risk of MI

**Figure 2:**
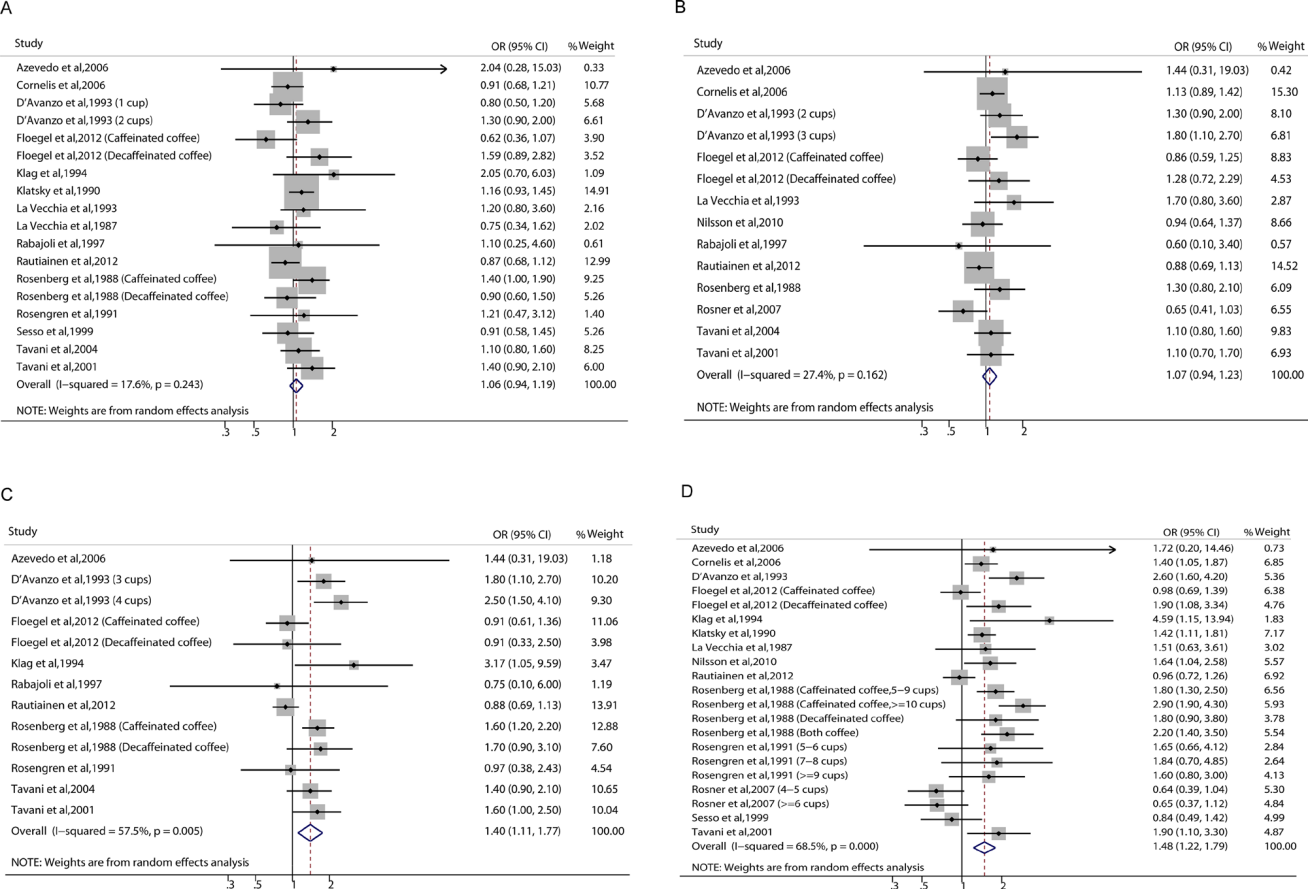
Relative risk of myocardial infarction associated with coffee consumption 1~2 cups (**A**), 2~3 cups (**B**), 3~4 cups (**C**), and > 4 cups (**D**) of coffee consumption per day in comparison with consumption of less than 1 cup per day.

The association between coffee consumption and risk of MI was not significant when comparing 1~2 cups with less than 1 cup per day of coffee consumption (OR = 1.06, 95% CI: 0.94–1.19) (Figure 2A). The pooled analysis showed no potential heterogeneity between the individual studies (I^2^ = 17.6, *p* = 0.243). In the subgroup analyses, the non-significant association was not changed by different sexes, coffee types, study locations, study designs, and NOS scores (Table 1). Moreover, sensitivity analysis suggested that the non-significant association was not changed by omitting one study in turn, with pooled ORs range from 1.03 (95% CI: 0.92–1.15) to 1.09 (95% CI: 0.97–1.23) (Supplementary Figure 1A).

**Table 1:** Subgroup analyses of relative risk of myocardial infarction associated with consumption of 1-2 cups, 2-3 cups, 3-4 cups, and over 4 cups of coffee per day in comparison with consumption of less than 1 cup per day (reference group), by sex, coffee type, study location, and study design

	Coffee Consumption, cups/day											
	1~2			2~3			3~4			> 4		
	Number of estimates	OR	95% CI	Number of estimates	OR	95% CI	Number of estimates	OR	95% CI	Number of estimates	OR	95% CI
Sex												
Men	8	1.16	0.96, 1.40	7	1.15	0.88, 1.50	8	1.75	1.44, 2.14	12	2.01	1.71, 2.36
Women	4	0.94	0.78, 1.14	6	0.91	0.74, 1.13	2	1.07	0.68, 1.68	6	0.91	0.67, 1.23
Both sexes	6	1.05	0.85, 1.31	6	1.00	0.87, 1.16	3	1.14	0.75, 1.74	8	1.35	1.14, 1.61
Coffee type												
Caffeinated	4	0.96	0.70, 1.31	2	1.03	0.80, 1.33	2	1.23	0.71, 2.13	5	1.45	0.99, 2.12
Decaffeinated	3	1.15	0.81, 1.63	2	1.42	0.90, 2.25	2	1.42	0.82, 2.47	2	1.86	1.19, 2.90
All types	11	1.07	0.95, 1.21	11	1.03	0.86, 1.22	9	1.49	1.07, 2.07	14	1.46	1.13, 1.88
Study location												
Europe	12	1.03	0.88, 1.22	13	1.02	0.87, 1.20	10	1.29	0.98, 1.71	13	1.32	1.02, 1.72
United States	5	1.15	0.96, 1.38	1	1.13	0.89, 1.43	3	1.68	1.29, 2.19	7	1.80	1.33, 2.45
Study design												
Cohort	6	1.05	0.81, 1.36	4	0.87	0.72, 1.05	5	0.96	0.74, 1.24	10	1.19	0.91, 1.55
Case-control	12	1.08	0.95, 1.22	11	1.14	0.98, 1.32	8	1.68	1.41, 1.99	11	1.79	1.45, 2.20
NOS score												
9	10	1.00	0.84, 1.19	10	1.09	0.91, 1.30	7	1.33	0.93, 1.90	10	1.24	0.93, 1.65
8	2	1.52	0.75, 3.10	0	-	-	2	1.69	0.53, 5.36	4	1.89	1.22, 2.93
7	6	1.14	0.98, 1.31	5	0.98	0.80, 1.20	4	1.54	1.23, 1.93	7	1.71	1.32, 2.22

OR = odds ratio, CI = confidential interval, NOS = Newcastle-Ottawa Scale.

Compared with less than 1 cup, 2~3 cups per day of coffee consumption was also non-significantly associated with the risk of MI (OR = 1.07, 95% CI: 0.94–1.23) (Figure 2B). The heterogeneity between the included studies was not significant (I^2^ = 27.4, *p* = 0.162). There was still no significant association in the subgroup analyses of sex, coffee type, study location, study design, and NOS score (Table 1). The sensitive analysis did not significantly change the results by omitting one study in turn, and pooled results of OR range from 1.01 (95% CI: 0.89–1.13) to 1.07 (95% CI: 0.95–1.22) (Supplementary Figure 1B).

Compared with less than 1 cup, 3~4 cups per day of coffee consumption significantly increased the risk of MI (OR = 1.40, 95% CI: 1.11–1.77) (Figure 2C), and there was potential heterogeneity between the individual studies (I^2^ = 57.5, *p* = 0.005). In the subgroup analyses, the positive association between coffee consumption and risk of MI was only found in the pooling result for men (OR = 1.75, 95% CI: 1.44–2.14), but not for women (OR = 1.07, 95% CI: 0.68–1.68) or both sexes (OR = 1.14, 95% CI: 0.75–1.74) (Table 1). The interaction for sex and the risk of MI was significant (*p* = 0.02). Moreover, this significant association was only found in studies that used all types of coffee consumption as the exposure of interest but not in studies that used caffeinated coffee or decaffeinated coffee, and in studies with NOS score of 7 but not in studies with NOS score of 9 and 8 (Table 1). The significantly increased risk was not observed in studies which were from Europe (OR = 1.29, 95% CI: 0.98–1.71) or which used a cohort design (OR = 0.96, 95% CI: 0.74–1.24) (Table 1). The interaction analyses for location (*p* = 0.28) and design (*p* = 0.09) were non-significant. The sensitive analysis did not significantly change the results by omitting one study in turn, and pooled results of OR range from 1.31 (95% CI: 1.05–1.63) to 1.51 (95% CI: 1.24–1.84) (Supplementary Figure 1C).

Compared with less than 1 cup, > 4 cups per day of coffee consumption had a significantly higher risk of MI (OR = 1.48, 95% CI: 1.22–1.79) (Figure 2D), with evidence of significant heterogeneity (I^2^ = 68.5, *p* < 0.001). Similarly, the significantly association was not observed in studies that included only women in the subgroup analysis (OR = 0.91, 95% CI: 0.67–1.23) (Table 1). The interaction for sex and the risk of MI was significant (*p* = 0.04). In addition, the higher risk was not significant in the studies that used caffeinated coffee as the exposure of interest, in studies that used a cohort design, or in studies with NOS score of 9 (Table 1). The interaction analyses for coffee type (*p* = 0.33), design (*p* = 0.26), and study quality (*p* = 0.41) were non-significant. The sensitive analysis did not significantly change the results by omitting one study in turn, and pooled results of OR range from 1.41 (95% CI: 1.18–1.70) to 1.55 (95% CI: 1.29–1.86) (Supplementary Figure 1D).

### Dose-response meta-analysis

**Figure 3:**
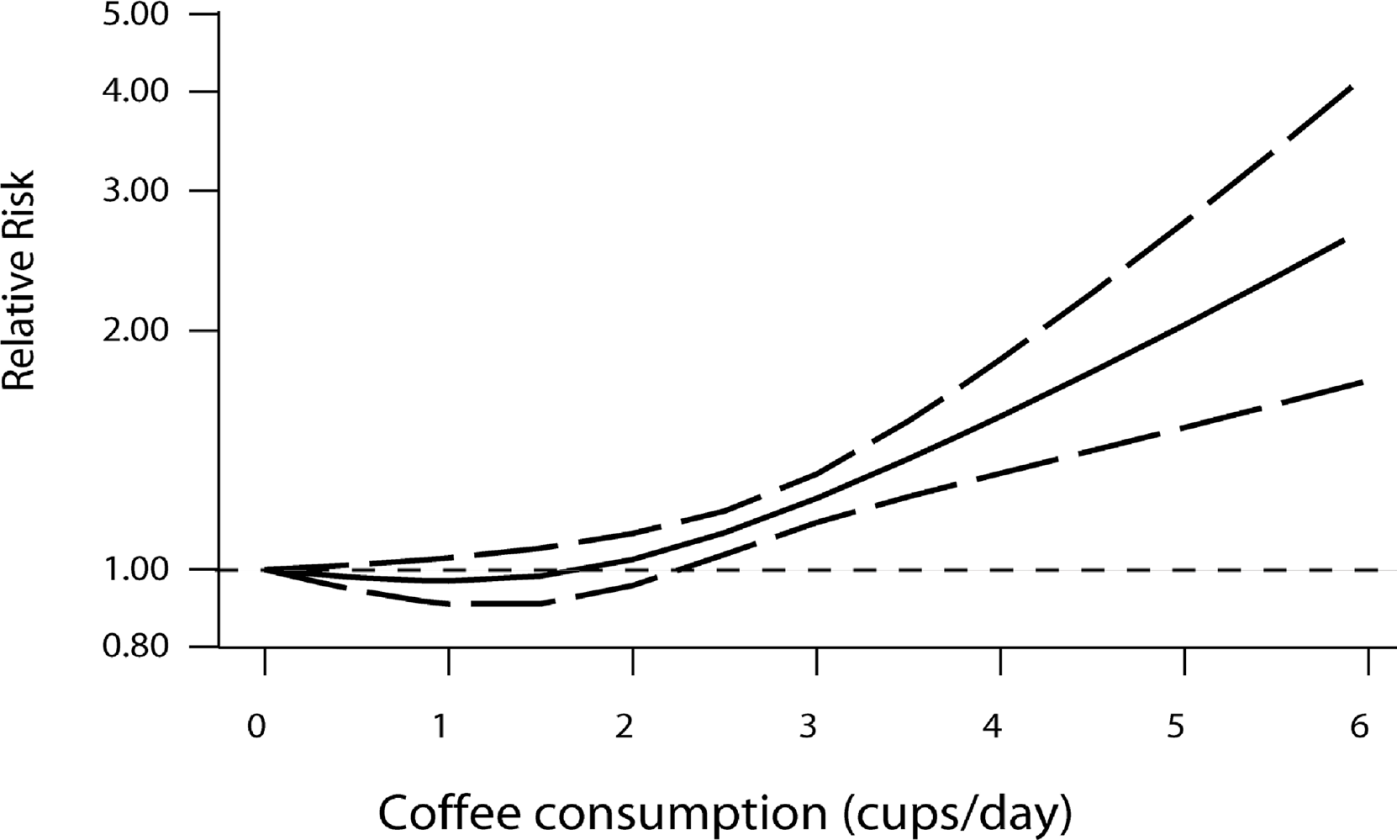
Dose-response relationship between coffee consumption and risk of myocardial infarction (**A)** generalized least squares trend estimation model was used to compute the specific slopes. –– and --- represent the estimated relative risks and 95% CIs.

As the meta-analysis included studies reporting the relative risks with their 95% CIs of MI relating to three or more categories of coffee consumption, all included studies were eligible to be included in the dose-response analysis. Coffee consumption and the risk of MI showed a non-linear relationship with statistical significance. (*p* = 0.034). The best fitting model showed a “J-shaped” curve (Figure 3).

### Publication bias

**Figure 4:**
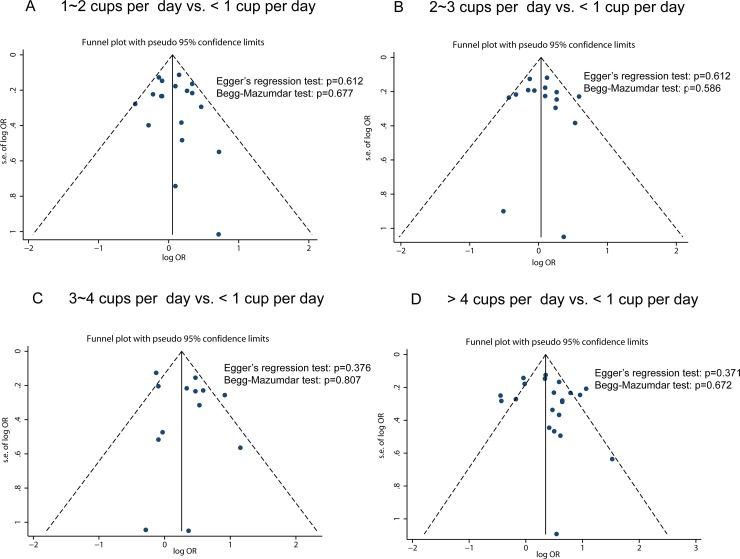
Funnel plots to explore publication bias The vertical line is at the mean effect size.

The studies were distributed fairly symmetrically about the combined effect size in funnel plots (Figure 4), which indicates that there was little potential publication bias. Egger's regression test (all *p* values > 0.05) and Begg-Mazumdar test (all *p* values > 0.05) also suggested that no publication bias was observed in the meta-analyses.

## DISCUSSION

Our study included 17 articles involving 233,617 participants and 10,510 patients with MI. Compared with less than 1 cup, coffee consumption of 1~2 cups and 2~3 cups per day did not significantly increased with the risk of MI; however, coffee consumption of 3~4 cups and > 4 cups per day was positively related with the risk of MI. This means that low to moderate coffee intake was not associated with an increased risk of MI, but heavy or very heavy intake significantly increase the risk. A dose-response relationship was observed between coffee consumption and the risk of MI, with a “J-shaped” relationship. Furthermore, the positive association was not changed in the sensitivity analyses and there was no publication bias in all the pooled results. Current dietary guidelines recommend moderate coffee intake [32], which were consistent with our results. Moreover, coffee consumption was also recommended to be restricted to1~2 cups per day by the American Heart Association [32].

Previous meta-analyses have assessed the association between coffee drinking and coronary heart disease risk [6, 33, 34]. There was also a meta-analysis examining the relationship between coffee intake and risk of death from MI and coronary disease [35]. Ding et al. [15] also performed a dose-response meta-analysis on coffee consumption and overall CVD risk. They found that CVD risk was reduced by moderate coffee consumption, and 3 to 5 cups per day of coffee consumption had the lowest CVD risk; however, there was no significant association between heavy coffee consumption and CVD risk. A recent meta-analysis [13] has evaluated the relationship between coffee and risk of death after acute MI and found that heavy coffee drinkers had a significantly lower risk of mortality after acute MI compared with both noncoffee drinkers and light coffee drinkers. Our results were inconsistent with the results they had found. This might be due to the fact that they used the overall CVD incidence or mortality after acute MI as the outcome; however, our study focused on the risk of MI incidence. In addition, the participants and methodological methods used in their meta-analyses might be different from our study. As far as we know, the relationship between coffee consumption and the risk of MI has not been concluded in a meta-analysis. In this latest meta-analysis, we included both cohort studies and case-control studies, because cohort and case-control studies tend to have complementary defects. Cohort studies assess the exposure limited to a few points in time; therefore, it is likely to occur nondifferential misclassification and blurs the association with the disease [28]. In contrast, case-control studies are likely to be affected by recall bias, and this may lead to differential misclassification of exposure and overestimation of the association. Case-control studies, in fact, can provide more accurate information on coffee consumption a short time before the occurrence of MI than can cohort studies, which generally obtain data on coffee consumption years or even decades before MI onset.

The most of individual studies only included male participants or female participants, and although some studies included both men and women, few studies have conducted stratified analysis by sex. In our meta-analysis, we conducted a subgroup analysis by sex and assessed the impact of sex on the pooled results. In the subgroup analyses by sex, we found that 1~2 cups and 2~3 cups per day of coffee consumption did not increase the risk of MI in both men and women, comparing with less than 1 cup per day; however, the point estimates were over 1 for men (1.16 and 1.15, respectively) and below 1 for women (0.94 and 0.91, respectively). In addition, 3~4 cups and > 4 cups per day of coffee consumption significantly increased the risk of MI in men (OR = 1.75, 95% CI: 1.44–2.14 and OR = 2.01, 95% CI: 1.71–2.36, respectively) but not in women (OR = 1.07, 95% CI: 0.68–1.68 and OR = 0.91, 95% CI: 0.67–1.23, respectively), comparing with less than 1 cup per day. Moreover, the interaction for sex and the risk of MI was significant. These results indicated that MI induced by coffee consumption is more serious for men than for women. This may be due to the fact that the number of estimates for women is less than men, which leads to a low statistical power. Other studies also reported different results on coffee consumption and CVD risk by different genders [34, 36, 37]. However, the reason why this relationship is different between different genders remains unknown. One possible reason is that smoking is strongly related to coffee drinking, and the proportion of male smokers is much higher than that of female smokers. One study reported very similar relative risks for coronary heart diseases at all categories of coffee consumption between male and female non-smokers [38]. Moreover, many studies have indicated that smoking is associated with unhealthy lifestyles (eg, physical inactivity, alcohol abuse) [29, 39]. Although most of the included studies in our meta-analysis had made adjustment for smoking, not all smoking-related unhealthy lifestyles were adjusted for. Thus, the gender differences in the association between coffee consumption and MI risk may be partly explained by smoking status and the related lifestyle habits. Further studies are still needed to evaluate the impact of gender on the association between coffee consumption and risk of MI.

A rapid increase in the risk of stroke and MI right after coffee ingestion has been reported in some studies [8, 40]. Caffeine, diterpenoids (primarily kahweol and cafestol), and polyphenols, the main components of coffee, have been reported to increase the risk of hypertension, serum concentrations of cholesterol and homocysteine, and the incidence of type 2 diabetes mellitus [41, 42]. A previous study reported that systolic blood pressure and diastolic blood pressure increased by 3~14 mm Hg and 2~13 mm Hg, respectively, after intake of caffeine equivalent to 2~3 cups of coffee in normotensive subjects [43]. Cornelis et al. [23] found that the positive association between coffee consumption and nonfatal MI was only significant in subjects with slow caffeine metabolism, suggesting that caffeine is a key in this association. Caffeine is reported to block the A_1_ and A_2A_ adenosine receptors [44, 45]. Adenosine is a potent coronary and systemic vasodilator, which may play an important role in the reactivity of inflammatory cells and platelets during myocardial ischemia [46, 47]. In the subgroup analysis of our study, we found the association with a higher risk of MI for moderate or heavy coffee consumption was only significant in studies that used all types of coffee and not in studies that used caffeinated coffee. This may due to the fact that the number of included studies in the subgroup of caffeinated coffee is limited (no more than five studies), leading to a low statistical power. Diterpenoids are present in the lipid fraction of boiled coffee, and have been shown to increase the serum cholesterol levels [48–50] and may increase the risk of MI. In addition, coffee consumption has an effect on total homocysteine levels in the general population. A cross-sectional study [51] has reported a positive association between homocysteine concentrations and coffee intake in a dose-dependent manner and this association has been confirmed in randomized controlled trials [7, 52]. Increased concentrations of total serum homocysteine have been reported to increase the risk of cardiovascular disease [1, 53]. The association between coffee consumption and MI risk is still controversial, and one possible explanation is that the polymorphic CYP1A2 enzyme is involved in the caffeine metabolism. Enzyme inducibility decreases when an A to C substitution at position 734 (CYP1A2^*^1F) in the *CYP1A2* gene, and the caffeine metabolism is impaired [54]. Therefore, “rapid” caffeine metabolizers are carriers of homozygous CYP1A2^*^1A allele and “slow” caffeine metabolizers are carriers of the variant CYP1A2^*^1F. One study [23] found that coffee drinking increased the risk of MI only in carriers of the ^*^1F allele but not in those with the ^*^1A/^*^1A genotype. Moreover, heavier coffee consumption may reflect increased mental stress level, which is also an important risk factor for the incidence of MI. However, further studies are needed on mechanism of coffee consumption and risk of MI.

The evidence from epidemiological studies on whether coffee consumption is a risk factor for MI is still sparse. The current study is the largest meta-analysis that investigates the dose-response relationship between coffee consumption and risk of MI. The strengths of the current study include: (1) we made a quantitative evaluation on how coffee consumption may influence risk of MI, based on the best available evidence from observational studies; (2) we combined all available dose in our dose-response meta-analysis across a large range of exposure, and the validity of the dose-response estimates have been increased; (3) the included studies have moderate-to-high quality; (4) the results from each individual studies has made maximum adjustment for confounding factors; (5) a relatively conservative conclusion but not an exaggerated one was obtained by using random-effects model to consider the heterogeneity between studies; and (6) our meta-analyses have no evidence of publication bias.

However, some limitations warrant consideration. First, potential heterogeneity was found in some of the results, and differences in study populations, study designs, determination methods of coffee consumption and MI, and methods of data analyses may contribute to the heterogeneity. We are unlikely to have fully accounted for the heterogeneity, although random-effects and sub-group analyses were conducted. Second, coffee drinking may, in fact, be a marker for sedentary life or atherogenic diet, most important, of cigarette smoking. Although most of included studies adjusted for known risk factors for risk of MI, such as sex, age, education, body mass index (BMI), alcohol drinking, smoking, diabetes mellitus, and hypertension, the possible bias from residual or unmeasured confounders remained. Therefore, caution should be taken when explaining our results. Third, all the included studies except one were from Europe or United States, studies from other regions, such as Asia, Africa, and Oceania, are unavailable. Therefore, the conclusions from this meta-analysis cannot be generalized to populations in other regions. Fourth, the type of processing and roasting of the coffee beans (e.g. higher level of roasting implies lower caffeine levels), the methods of coffee preparation (e.g., filtered, boiled, espresso), and the definition of “1 cup” were not reported in most of included studies; therefore, we cannot evaluate the impact of these information on the results. Finally, we cannot confirm a causal relationship between coffee consumption and risk of MI since our results are based on evidence from observational studies.

In conclusion, our meta-analysis included17 independent observational studies, which indicated that daily drinking of 3~4 cups and > 4 cups of coffee were positively related to the risk of MI, compared with less than 1 cup. However, this positive association was only found in men but not in women. We found a “J-shaped” association between coffee consumption and risk of MI. In light of the widespread use of coffee, this adverse effect should be further investigated, and it would be advisable to avoid the heavy consumption of coffee. As the age of participants in the included studies were about 50–60 years old and only men were tightly linked to heavy coffee consumption and MI in the meta-analysis, the results must be interpreted with caution in other age groups and more studies are needed to further evaluate the impact of gender on the association between coffee consumption and risk of MI.

## MATERIALS AND METHODS

We strictly followed the guidelines published by the Meta-analysis of Observational Studies in Epidemiology (MOOSE) group to complete the meta-analysis [55].

### Study selection

Eligible articles were searched in electronic databases of Medline, Embase, and Cochrane Library (from 1970 to Feb 9, 2017) by two investigators. The search strategy included both MeSH and free-text terms. The following terms were used: “Coffee [MeSH]”, “Myocardial Infarction [MeSH]”, “coffee”, “coffee intake”, “coffee consumption”, and “myocardial infarction”. The search strategy was limited by study design, and only cohort studies and case-control studies were included. The references of all retrieved articles and recent review were also manually screened. No attempt was made to find articles in languages other than English. We contacted the authors for additional data when necessary.

A study was eligible for inclusion if the following criteria were met: (1) a cohort or case-control design; (2) examination of coffee consumption as the exposure of interest; (3) determination of MI as the outcome of interest; and (4) reporting the relative risks with their 95% confidence intervals (CIs) of MI relating to three or more categories of coffee consumption. The studies about review research, animal experiment, and mechanistic research were excluded.

### Data extraction and study quality evaluation

Two investigators extracted the data independently using a predefined data collection form, with disagreements being resolved by consensus. Relevant data included the first author's surname, publication year, location of the study, study design, gender of the participants, age of the participants, sample size of the study, number of MI events, ascertainment method of coffee consumption, ascertainment method of MI, coffee type, coffee consumption categories, most fully adjusted relative risks and corresponding 95% CIs for every category of coffee consumption, and covariates adjusted in the multi-variable analysis.

The quality of each included study was assessed by two investigators, using the Newcastle-Ottawa Scale (NOS) recommended by Wells and colleagues [56]. The NOS score of each included study ranges from 0 to 9 points, and higher points indicated higher study quality. The following domains were assessed according to the NOS: the basis of the participant selection (0-4 points), the comparability of the cohort or case-control design and analysis (0-2 points), and the adequacy of measurements including exposure and outcome variables (0-3 points).

### Statistical analysis

Data was classified into five categories of coffee consumption as follows: < 1 cup per day, 1~2 cups per day, 2~3 cups per day, 3~4 cup per day and > 4 cups per day. An approach of generic inverse-variance was used to pool the outcome data for the coffee consumption categories of 1~2 vs. < 1 cup per day, 2~3 vs. < 1 cup per day, 3~4 vs. < 1 cup per day and > 4 vs. < 1 cup per day with a random-effects model. I^2^ statistic was used to examine the heterogeneity between the studies (I^2^ > 50% was indicated as significant heterogeneity) [57]. Subgroup analyses were performed according to the sex, coffee type, study location, study design, and NOS score. We also conducted sensitivity analyses by removing each individual study from the meta-analyses to test the effect of an individual article on the whole combined results. Funnel plots were used to visually assess the publication bias. Egger's regression test [58] and Begg-Mazumdar test [59] were used to further assess publication bias.

In the dose-response analysis, a generalized least squares trend estimation model was used to compute the specific slopes [60, 61]. For categories (at least three) of coffee consumption that were open (e.g., 1~2 cups per day), the median value was assigned as the homologous category of coffee consumption. If the maximum dose was fixed unlimitedly (e.g., > 4 cups per day), we assumed that the mean was 25% larger than the lower level of the specific category [42]. The results of dose-response analysis were shown for each cup increased in daily coffee drinking. We used a restricted cubic spline model (four-knot) to test the non-linearity hypothesis in the relation of coffee intake with the occurrence of MI.

Statistical analyses were conducted using the Stata (version 12.0). All *P* values less than 0.05 were considered as significant.

## SUPPLEMENTARY MATERIALS FIGURE AND TABLES








