# Supplementary material for: Coffee consumption and risk of myocardial infarction: a dose-response meta-analysis of observational studies
Source: Oncotarget. 2018 Jan 4;9(30):21530–40. doi: 10.18632/oncotarget.23947 (PMC5940396; doi:10.18632/oncotarget.23947)
Supplement: Supplementary file 1 [file oncotarget-09-21530-s001.pdf]

Coffee consumption and risk of myocardial infarction: a dose-response meta-analysis of observational studies

SUPPLEMENTARY MATERIALS

Supplementary Table 1: Quality assessment of the included studies (cohort studies). See_Supplementary_Table 1

Supplementary Table 2: Quality assessment of the included studies (case-control studies). See_Supplementary_Table 2

Supplementary Table 3: Characteristics of included studies. See_Supplementary_Table 3

A

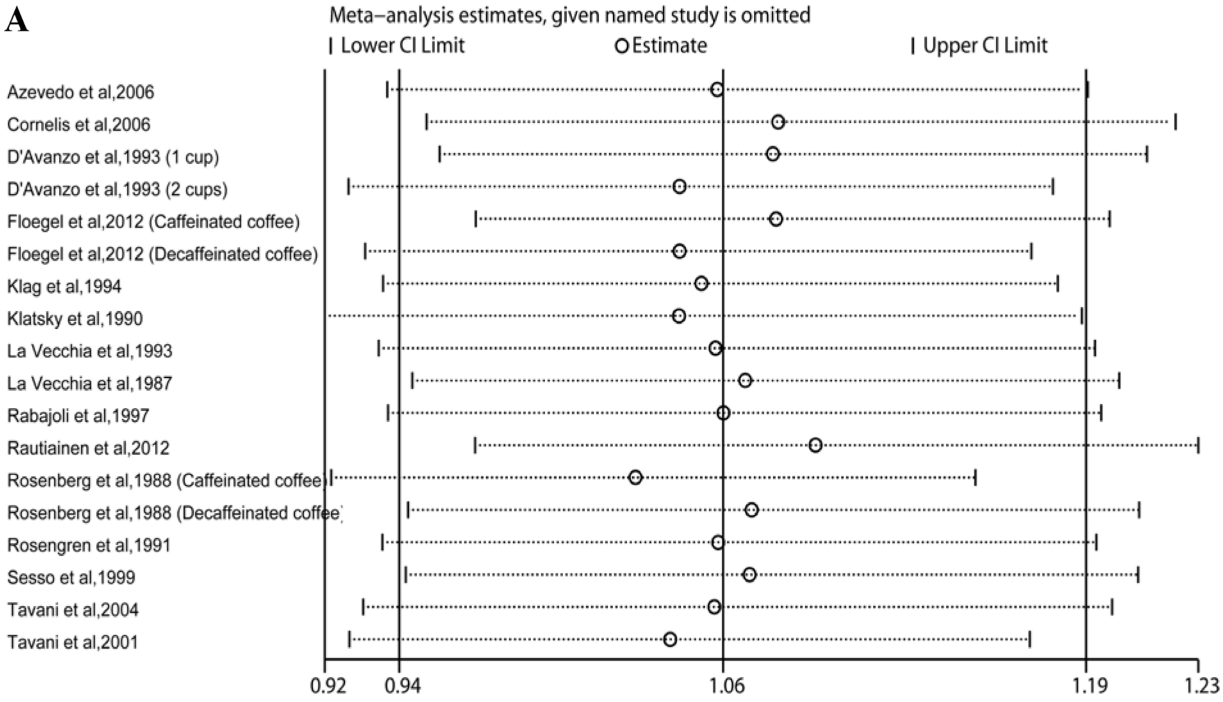
Meta−analysis estimates, given named study is omitted
Lower CI Limit
Estimate
Upper CI Limit
Azevedo et al,2006
Cornelis et al,2006
D'Avanzo et al,1993 (1 cup)
D'Avanzo et al,1993 (2 cups)
Floegel et al,2012 (Caffeinated coffee)
Floegel et al,2012 (Decaffeinated coffee)
Klag et al,1994
Klatsky et al,1990
La Vecchia et al,1993
La Vecchia et al,1987
Rabajoli et al,1997
Rautiainen et al,2012
Rosenberg et al,1988 (Caffeinated coffee
Rosenberg et al,1988 (Decaffeinated coffee
Rosengren et al,1991
Sesso et al,1999
Tavani et al,2004
Tavani et al,2001
0.92
0.94
1.06
1.19
1.23

B

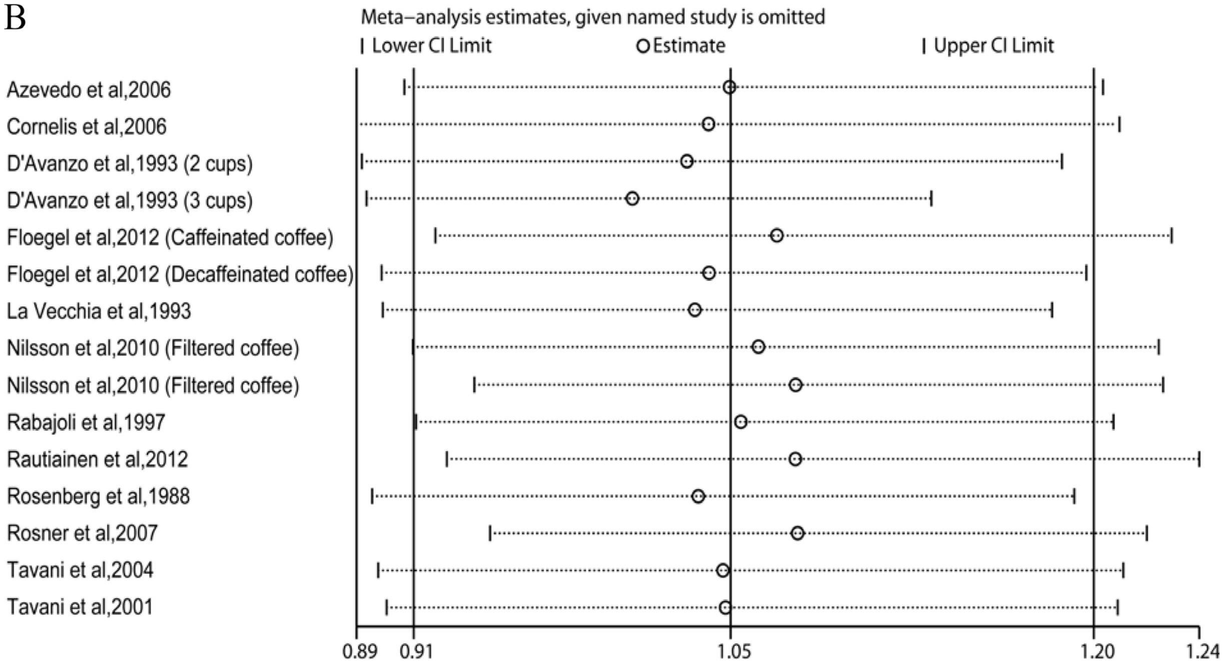
Meta−analysis estimates, given named study is omitted
Lower CI Limit
Estimate
Upper CI Limit
Azevedo et al,2006
Cornelis et al,2006
D'Avanzo et al,1993 (2 cups)
D'Avanzo et al,1993 (3 cups)
Floegel et al,2012 (Caffeinated coffee)
Floegel et al,2012 (Decaffeinated coffee)
La Vecchia et al,1993
Nilsson et al,2010 (Filtered coffee)
Nilsson et al,2010 (Filtered coffee)
Rabajoli et al,1997
Rautiainen et al,2012
Rosenberg et al,1988
Rosner et al,2007
Tavani et al,2004
Tavani et al,2001
0.89
0.91
1.05
1.20
1.24

C

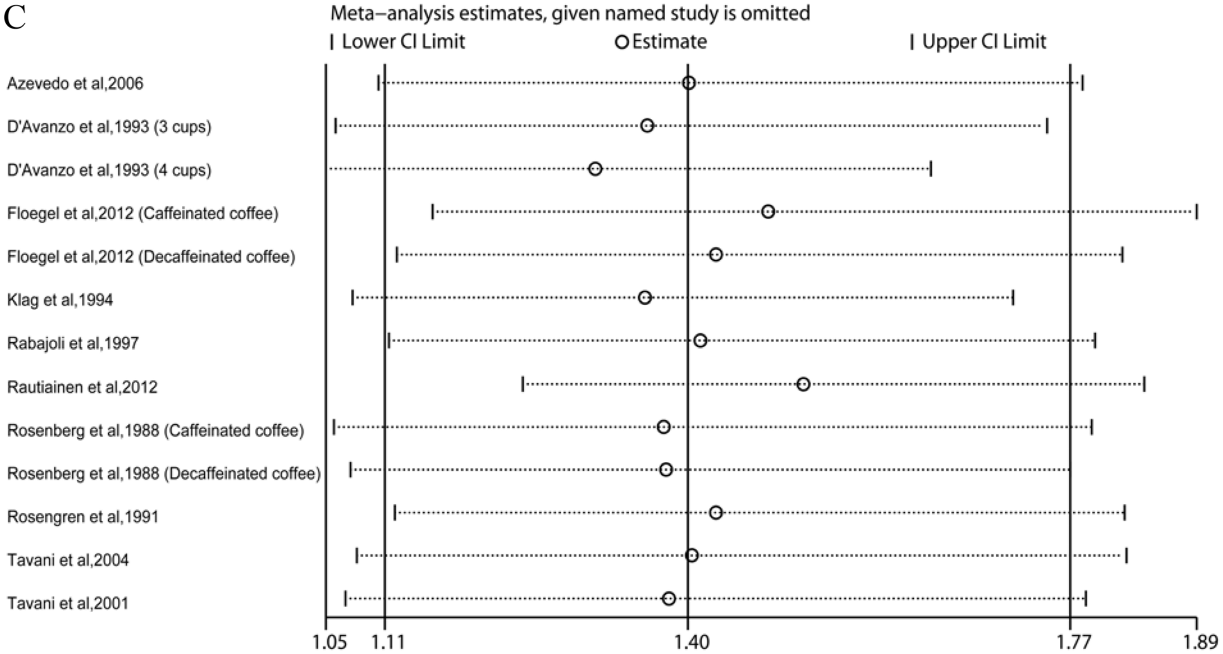

D

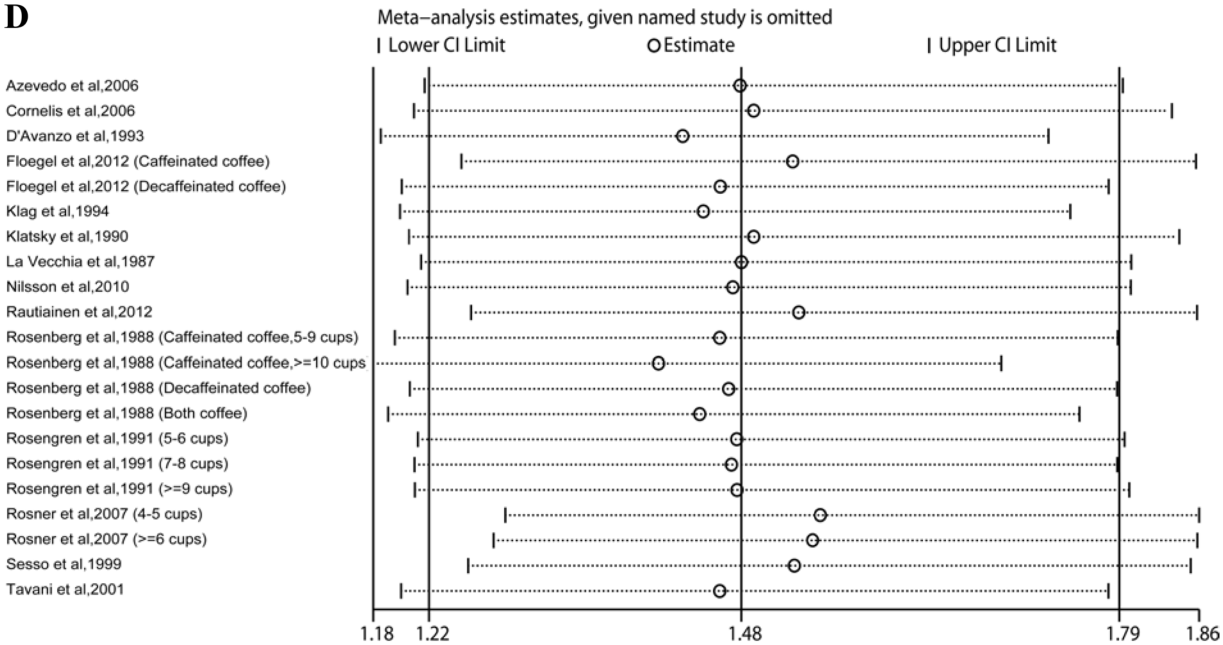

Supplementary Figure 1: Sensitivity analysis. Relative risk of myocardial infarction associated with consumption of 1-2 cups (**A**), 2-3 cups (**B**), 3-4 cups (**C**), and over 4 cups (**D**) of coffee per day in comparison with consumption of less than 1 cup per day by omitting one study in turn.
