# Supplementary material for: Coffee consumption and risk of myocardial infarction: a dose-response meta-analysis of observational studies
Source: Oncotarget. 2018 Jan 4;9(30):21530–40. doi: 10.18632/oncotarget.23947 (PMC5940396; doi:10.18632/oncotarget.23947)
Supplement: Supplementary file 2 [file oncotarget-09-21530-s002.doc]

**Supplementary Table 1: Quality assessment of the included studies (cohort studies)**

| Study | Selection | | | | Comparability | Outcome | | | Overall quality assessment score (of a maximum of 9) |
| --- | --- | --- | --- | --- | --- | --- | --- | --- | --- |
|  | Representativeness of the exposed cohort | Selection of the non exposed cohort | Ascertainment of exposure | Demonstration that outcome of interest was not present at start of study | Comparability of cohorts on the basis of the design or analysis | Assessment of outcome | Was follow-up long enough for outcomes to occur | Adequacy of follow up of cohorts |  |
| Dai et al, 2006 | *Truly representative of the average population in the community | * Drawn from the same community as the exposed cohort | * Structured interview | *The study demonstrated that MI was not present at start of study | ** Study controls for age, sex, smoking, alcohol intake, physical activity, education, employment, vitamin and mineral supplement use during past 4 wk, total energy intake, tea intake, caffeinated/decaffeinated coffee intake, BMI, waist-to-hip ratio, and prevalent hypertension | * Independent blind assessment | * The study selected an adequate follow up period for outcome of interest | *Subjects lost to follow up might not introduce bias (80%) follow up | 9 |
| Klag et al,1994 | *Truly representative of the average population in the community | * Drawn from the same community as the exposed cohort | * Structured interview | The study did not demonstrate that MI was not present at start of study | ** Study controls for age, baseline serum cholesterol, calendar time and time-dependent hypertension, number of cigarettes, diabetes, and BMI | * Independent blind assessment | * The study selected an adequate follow up period for outcome of interest | *Subjects lost to follow up might not introduce bias (100%) follow up | 8 |
| Klatsky et al,1990 | *Truly representative of the average population in the community | * Drawn from the same community as the exposed cohort | * Structured interview | The study did not demonstrate that MI was not present at start of study | ** Study controls for age, sex, race, smoking, alcohol, education, and baseline disease | * Independent blind assessment | * The study selected an adequate follow up period for outcome of interest | Subjects lost to follow up might introduce bias (11%) follow up | 7 |
| Rautiainen et al,2012 | *Truly representative of the average population in the community | * Drawn from the same community as the exposed cohort | * Structured interview | *The study demonstrated that MI was not present at start of study | ** Study controls for age, education, smoking, BMI, physical activity, hypertension, hypercholesterolemia, family history of MI, aspirin use, hormone replacement therapy use, dietary supplement use, and intakes of total energy and alcohol | * Independent blind assessment | * The study selected an adequate follow up period for outcome of interest | *Subjects lost to follow up might not introduce bias (99%) follow up | 9 |
| Rosengren et al,1991 | *Truly representative of the average population in the community | * Drawn from the same community as the exposed cohort | * Structured interview | *The study demonstrated that MI was not present at start of study | ** Study controls for age, systolic blood pressure, BMI, diabetes, registration for alcohol abuse, family history of MI, mental stress, physical activity, and occupational class | * Independent blind assessment | * The study selected an adequate follow up period for outcome of interest | Subjects lost to follow up might introduce bias (68%) follow up | 8 |
| Rosner et al,2007 | *Truly representative of the average population in the community | * Drawn from the same community as the exposed cohort | * Structured interview | *The study demonstrated that MI was not present at start of study | ** Study controls for age, smoking status, total activity score, alcohol consumption, diabetes, hypercholesterolemia, hypertension, BMI, family history of MI before age 60 years, hormone replacement therapy use, multivitamin use, vitamin E supplement use, educational level, tea, sugar in tea or coffee, and quartiles of energy-adjusted folate, fiber, saturated fat, monounsaturated fat, and polyunsaturated fat | * Independent blind assessment | * The study selected an adequate follow up period for outcome of interest | *Subjects lost to follow up might not introduce bias (99%) follow up | 9 |
