# Supplementary material for: Coffee consumption and risk of myocardial infarction: a dose-response meta-analysis of observational studies
Source: Oncotarget. 2018 Jan 4;9(30):21530–40. doi: 10.18632/oncotarget.23947 (PMC5940396; doi:10.18632/oncotarget.23947)
Supplement: Supplementary file 3 [file oncotarget-09-21530-s003.doc]

**Supplementary Table 2: Quality assessment of the included studies (case-control studies)**

| Study | Selection | | | | Comparability | Exposure | | | Overall quality assessment score (of a maximum of 9) |
| --- | --- | --- | --- | --- | --- | --- | --- | --- | --- |
|  | Is the case definition adequate? | Representativeness of the cases | Selection of controls | Definition of Controls | Comparability of cases and controls on the basis of the design or analysis | Ascertainment of exposure | Same method of ascertainment for cases and controls | Non-Response rate |  |
| Azevedo et al,2006 | * Yes, with independent validation | * Somewhat representative of the average population in the community | * Drawn from the same community as the cases | * No history of disease | ** The study controls for family history of AMI, product-term of coffee and family history, age, education, smoking status and waist-to-hip ratio | * Secure record | * Yes | * Same rate for both groups | 9 |
| Cornelis et al,2006 | * Yes, with independent validation | * Truly representative of the average population in the community | * Drawn from the same community as the cases | * No history of disease | ** The study controls for Smoking, waist-hip ratio, income, physical activity, history of diabetes, history of hypertension and intakes of alcohol, total energy, and energy-adjusted saturated fat, polyunsaturated fat, trans fat, folate, and sucrose | * Secure record | * Yes | * Same rate for both groups | 9 |
| D'Avanzo et al,1993 | * Yes, with independent validation | * Truly representative of the average population in the community | * Drawn from the same community as the cases | * No history of disease | ** The study controls for age, education, marital status, area of residence, body mass index, smoking habits, alcohol consumption, family history for AMI, cholesterol level, history of diabetes, and hypertension | * Secure record | * Yes | * Same rate for both groups | 9 |
| La Vecchia et al,1993 | * Yes, with independent validation | * Truly representative of the average population in the community | * Drawn from the same community as the cases | * No history of disease | ** The study controls for age, marital status, education, regular-coffee consumption, alcohol consumption, BMI, smoking habits, diabetes, hypertension, and hyperlipidemia | * Secure record | * Yes | * Same rate for both groups | 9 |
| La Vecchia et al,1987 | * Yes, with independent validation | * Somewhat representative of the average population in the community | * Drawn from the same community as the cases | * No history of disease | ** The study controls for geographic area, marital status, education, social class, cigarette smoking, alcohol consumption, parity, age at menopause, diabetes, hypertension, obesity, hyperlipidemia, family history of ischemic heart disease, and oral contraceptive and other female hormone use | * Secure record | * Yes | * Same rate for both groups | 9 |
| Nilsson et al,2010 | * Yes, with independent validation | * Truly representative of the average population in the community | No description | * No history of disease | * The study controls for current smoking, postsecondary education, apolipoprotein B/apolipoprotein A-I ratio and BMI | * Secure record | * Yes | * Same rate for both groups | 7 |
| Rabajoli et al,1997 | * Yes, with independent validation | * Somewhat representative of the average population in the community | No description | * No history of disease | * The study controls for age and family history | * Secure record | * Yes | * Same rate for both groups | 7 |
| Rosenberg et al,1988 | No (only by telephone call) | * Truly representative of the average population in the community | No description | * No history of disease | ** The study controls for categories of caffeine-containing coffee consumption, decaffeinated coffee consumption, tea consumption, age, cigarette smoking, drug-treated hypertension, drug-treated diabetes mellitus, BMI, Framingham Type A personality score, hours per week of vigorous leisure-time physical activity, alcohol consumption, family history of MI, religion, years of education, year of interview, geographic area, and number of visits to a physician in the previous year | * Secure record | * Yes | * Same rate for both groups | 7 |
| Sesso et al,1999 | No description | * Somewhat representative of the average population in the community | * Drawn from the same community as the cases | No description | ** The study controls for age, sex, smoking status, history of medication for high blood pressure, type A personality, family history of MI, diabetes, daily aspirin use, BMI, log of physical activity index, percentage of calories from saturated fat, total caloric intake, and alcohol intake | * Secure record | * Yes | * Same rate for both groups | 7 |
| Tavani et al,2004 | No description | * Somewhat representative of the average population in the community | * Drawn from the same community as the cases | No description | ** The study controls for age, study, education, BMI, smoking, alcohol drinking, diabetes, byperlipidemia, hypertension, and family history of AMI in first-degree relatives | * Secure record | * Yes | * Same rate for both groups | 7 |
| Tavani et al,2001 | * Yes, with independent validation | * Somewhat representative of the average population in the community | * Drawn from the same community as the cases | * No history of disease | ** The study controls for age, sex, education, BMI, cholesterol, smoking, alcohol drinking, physical activity, hyperlipidemia, diabetes, hypertension and family history of AMI in first degree relatives, and tea drinking | * Secure record | * Yes | * Same rate for both groups | 9 |
