# Supplementary material for: Coffee consumption and risk of myocardial infarction: a dose-response meta-analysis of observational studies
Source: Oncotarget. 2018 Jan 4;9(30):21530–40. doi: 10.18632/oncotarget.23947 (PMC5940396; doi:10.18632/oncotarget.23947)
Supplement: Supplementary file 4 [file oncotarget-09-21530-s004.doc]

**Supplementary Table 3**. Characteristics of included studies

| Study | Location | Study design | Sex | Sample size | Number of disease | Age, mean/range | Exposure variable ascertainment | Exposure variable | MI ascertainment | Adjustment | NOS |
| --- | --- | --- | --- | --- | --- | --- | --- | --- | --- | --- | --- |
| Azevedo et al, 2006 | Portugal | Case-control | Men | 654 | 290 | 58.8 | SAQ | Coffee | Hospital diagnosis | Family history of AMI, product-term of coffee and family history, age, education, smoking status and waist-to-hip ratio | 9 |
| Cornelis et al, 2006 | Costa Rica | Case-control | Men and women | 4028 | 2014 | 58.3 | FFQ | Caffeinated coffee | WHO criteria for MI plus either cardiac enzyme levels or electrocardiogram | Smoking, waist-hip ratio, income, physical activity, history of diabetes, history of hypertension and intakes of alcohol, total energy, and energy-adjusted saturated fat, polyunsaturated fat, trans fat, folate, and sucrose | 9 |
| D'Avanzo et al, 1993 | Italy | Case-control | Men | 1593 | 801 | 56 | SAQ | Coffee | Standard criteria | Age, education, marital status, area of residence, BMI, smoking habits, alcohol consumption, family history for AMI, cholesterol level, history of diabetes, and hypertension | 9 |
| Floegel et al, 2012 | Germany | Cohort | Men and women | 42659 | 394 | 49.8 | FFQ | Caffeinated coffee; Decaffeinated coffee | ICD-10 | Age, sex, smoking, alcohol intake, physical activity, education, employment, vitamin and mineral supplement use during past 4 wk, total energy intake, tea intake, caffeinated/decaffeinated coffee intake, BMI, waist-to-hip ratio, and prevalent hypertension | 9 |
| Klag et al, 1994 | United States | Cohort | Men | 933 | 61 | 26.3 | SAQ | Coffee | ICD-9 | Age, baseline serum cholesterol, calendar time and time-dependent hypertension, number of cigarettes, diabetes, and BMI | 8 |
| Klatsky et al, 1990 | United States | Cohort | Men and women | 101774 | 740 | NR | SAQ | Coffee | ICD-8 | Age, sex, race, smoking, alcohol, education, and baseline disease | 7 |
| La Vecchia et al, 1993 | Italy | Case-control | Women | 1302 | 433 | 52 | SAQ | Decaffeinated coffee | Standard WHO criteria for MI | Age, marital status, education, regular-coffee consumption, alcohol consumption, BMI, smoking habits, diabetes, hypertension, and hyperlipidemia | 9 |
| La Vecchia et al, 1987 | Italy | Case-control | Women | 419 | 168 | 47.4 | SAQ | Coffee | Standard WHO criteria for MI | Geographic area, marital status, education, social class, cigarette smoking, alcohol consumption, parity, age at menopause, diabetes, hypertension, obesity, hyperlipidemia, family history of ischemic heart disease, and oral contraceptive and other female hormone use | 9 |
| Nilsson et al, 2010 | Sweden | Case-control | Men and women | 1668 | 375 | 54 | FFQ | Coffee | Standard WHO criteria for MI | Current smoking, postsecondary education, apolipoprotein B/apolipoprotein A-I ratio and BMI | 7 |
| Rabajoli et al, 1997 | Italy | Case-control | Men | 310 | 153 | NR | FFQ | Coffee | Clinical history, electrocardiogram, and/or serum CK and CK-MB | Age and family history | 7 |
| Rautiainen et al, 2012 | Sweden | Cohort | Women | 32561 | 1114 | 61 | FFQ | Coffee | Swedish Hospital Discharge Registry and the Swedish Causes of Death Registry | Age, education, smoking, BMI, physical activity, hypertension, hypercholesterolemia, family history of MI, aspirin use, hormone replacement therapy use, dietary supplement use, and intakes of total energy and alcohol | 9 |
| Rosenberg et al, 1988 | United States | Case-control | Men | 3034 | 1873 | 43.7 | SAQ | Caffeinated coffee; Decaffeinated coffee; Both | Discharge summary | Categories of caffeine-containing coffee consumption, decaffeinated coffee consumption, tea consumption, age, cigarette smoking, drug-treated hypertension, drug-treated diabetes mellitus, BMI, Framingham Type A personality score, hours per week of vigorous leisure-time physical activity, alcohol consumption, family history of MI, religion, years of education, year of interview, geographic area, and number of visits to a physician in the previous year | 7 |
| Rosengren et al, 1991 | Sweden | Cohort | Men | 6765 | 230 | 51-59 | SAQ | Coffee | ICD-8 | Age, systolic blood pressure, BMI, diabetes, registration for alcohol abuse, family history of MI, mental stress, physical activity, and occupational class | 8 |
| Rosner et al, 2007 | Sweden | Cohort | Women | 32650 | 459 | 60.7 | FFQ | Coffee | Swedish Hospital Discharge Register and the Swedish Causes of Death Registry. | Age, smoking status, total activity score, alcohol consumption, diabetes, hypercholesterolemia, hypertension, BMI, family history of MI before age 60 years, hormone replacement therapy use, multivitamin use, vitamin E supplement use, educational level, tea, sugar in tea or coffee, and quartiles of energy-adjusted folate, fiber, saturated fat, monounsaturated fat, and polyunsaturated fat | 9 |
| Sesso et al, 1999 | United States | Case-control | Men and women | 680 | 340 | 57.7 | FFQ | Caffeinated coffee; Decaffeinated coffee | Serum CK and hospital discharge | Age, sex, smoking status, history of medication for high blood pressure, type A personality, family history of MI, diabetes, daily aspirin use, BMI, log of physical activity index, percentage of calories from saturated fat, total caloric intake, and alcohol intake | 7 |
| Tavani et al, 2004 | Italy | Case-control | Women | 1602 | 558 | 53.7 | SAQ | Coffee | WHO criteria for MI | Age, study, education, BMI, smoking, alcohol drinking, diabetes, hyperlipidemia, hypertension, and family history of AMI in first-degree relatives | 7 |
| Tavani et al, 2001 | Italy | Case-control | Men and women | 985 | 507 | 60 | SAQ | Coffee | ICD-9 | Age, sex, education, BMI, cholesterol, smoking, alcohol drinking, physical activity, hyperlipidemia, diabetes, hypertension and family history of AMI in first degree relatives, and tea drinking | 9 |

NOS= Newcastle-Ottawa Scale, SAQ= self-administered questionnaire, FFQ= food-frequency questionnaire, AMI= acute myocardial infarction, BMI= body mass index, MI= myocardial infarction, WHO= World Health Organization, ICD= International Statistical Classification of Diseases, CK= creatine phosphokinase
